# The hydro­chloride salt of 4-hy­droxy-*N*,*N*-di-*n*-propyl­tryptamine (4-HO-DPT)

**DOI:** 10.1107/S2414314620015461

**Published:** 2020-11-27

**Authors:** Vamshikrishna Reddy Sammeta, Sivappa Rasapalli, Andrew R. Chadeayne, James A. Golen, David R. Manke

**Affiliations:** a University of Massachusetts Dartmouth, 285 Old Westport Road, North Dartmouth, MA 02747, USA; b CaaMTech, LLC, 58 East Sunset Way, Suite 209, Issaquah, WA 98027, USA; University of Otago, New Zealand

**Keywords:** crystal structure, tryptamines, indoles, hydrogen bonding

## Abstract

The title compound, 4-hy­droxy-*N*,*N*-di-*n*-propyl­tryptammonium (4-HO-DPT) chloride {systematic name: *N*-[2-(4-hy­droxy-1*H*-indol-3-yl)eth­yl]-*N*-propyl­propan-1-aminium chloride}, C_16_H_25_N_2_O^+^·Cl^−^, has a singly protonated trypt­ammonium cation and one chloride anion in the asymmetric unit. A series of N—H⋯Cl and O—H⋯Cl hydrogen bonds connect the ions together in ladder chains along [010].

## Abstract 2

The first single-crystal structure of the hydro­chloride salt of a psilocin analogue, specifically 4-hy­droxy-*N*,*N*-di-*n*-propyl­tryptamine (4-HO-DPT) is reported.

## Graphical abstract

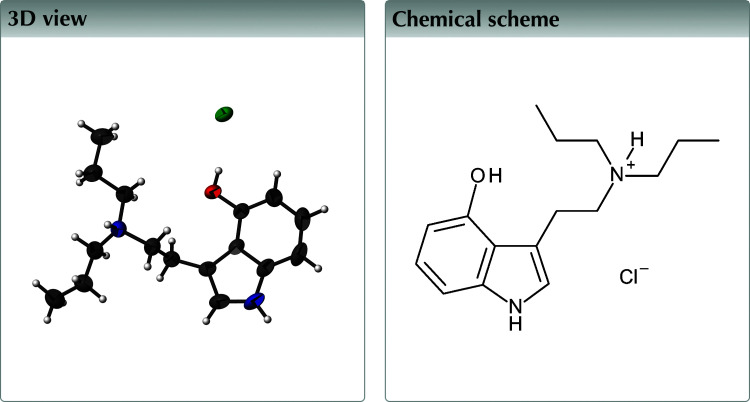

## Structure description

**Figure 1:**
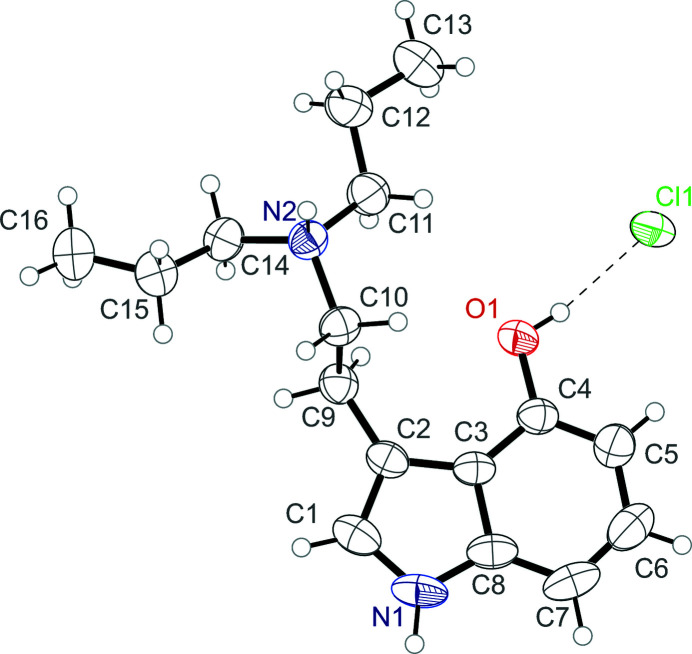
The mol­ecular structure of 4-hy­droxy-*N*,*N*-di-*n*-propyl­tryptammonium (4-HO-DPT) chloride, showing the atom labeling. Displacement ellipsoids are drawn at the 50% probability level. A hydrogen bond is shown as a dashed line.

Psilocybin, *N*,*N*-dimethyl-4-phospho­ryloxytryptamine, is a naturally occurring tryptamine found in ‘magic mushrooms’. When consumed orally, the phosphate group of psilocybin is hydrolyzed to generate psilocin, 4-hy­droxy-*N*,*N*-di­methyl­tryptamine. Psilocybin functions as a prodrug of psilocin, its active metabolite, which binds to and stimulates the human serotonin 2a receptor (Geiger *et al.*, 2018 ▸), causing profound changes to human consciousness, which are generally described as a ‘psychedelic’ experience. Recently, human serotonin 2a receptor agonists (*e.g.* LSD, ayahuasca/DMT, psilocybin/psilocin, peyote/mescaline, 5-MeO-DMT) have shown incredible potential for treating a wide variety of the world’s most debilitating, intra­ctable, and costly health problems, including anxiety, depression, addiction, post-traumatic stress disorder (PTSD) and inflammation (Johnson & Griffiths, 2017 ▸; Carhart-Harris & Goodwin, 2017 ▸). 4-Hy­droxy-*N*,*N*-di-*n*-propyl­tryptamine (4-HO-DPT) is a structural analogue of psilocin and produces similar (though not identical) psychedelic effects in human subjects (Shulgin & Shulgin, 2016 ▸). The compound 4-HO-DPT is somewhat more hydro­phobic than psilocin on account of substituting the di-*n*-methyl­amine group with a di-*n*-propyl­amine group. When used as pharmaceuticals, amines are often converted to their hydro­chloride salts to increase their solubility in water, and thus improving drug delivery (Stahl & Wermuth, 2011 ▸). Herein we report the solid-state structure of 4-hy­droxy-*N*,*N*-di-*n*-propyl­tryptammonium (4-HO-DPT) chloride (Fig. 1 ▸), which is the first of a psilocin analogue as its hydro­chloride salt.

**Figure 2:**
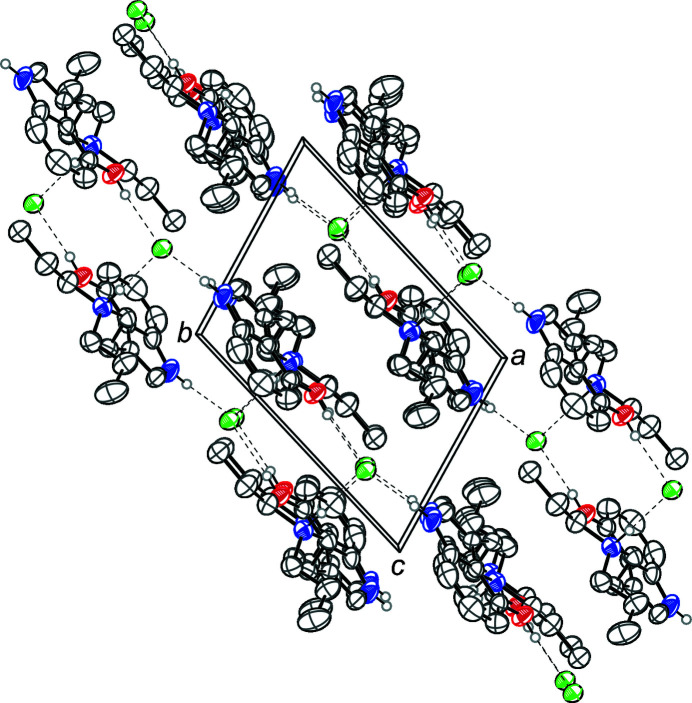
The crystal packing of 4-hy­droxy-*N*,*N*-di-*n*-propyl­tryptammonium (4-HO-DPT) chloride, viewed along the *c* axis. The hydrogen bonds (Table 1 ▸) are shown as dashed lines. Displacement ellipsoids are drawn at the 50% probability level. Hydrogen atoms not involved in hydrogen bonds are omitted for clarity.

The 4-HO-DPT cation and the chloride anion are held together in the asymmetric unit *via* O—H⋯Cl hydrogen bonds. The cation possesses a near planar indole group, with a mean deviation from planarity of 0.011 Å. The ethyl­amino group is turned away from the plane with a C1—C2—C9—C10 torsion angle of 101.54 (18)°. The N—H of the indole ring, and the N—H of the ammonium group also both hydrogen bond to other symmetry-generated chloride anions through N—H⋯Cl inter­actions (Table 1 ▸). The N—H⋯Cl indole-to-chloride hydrogen bond and the O—H⋯Cl hydrogen bond combine to link the ions together in chains with graph-set notation 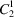 (8). The N—H⋯Cl indole-to-chloride hydrogen bonds and the N—H⋯Cl ammonium-to-chloride hydrogen bonds combine to generate rings with graph-set notation 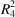 (18) (Etter, *et al.* 1990 ▸). The N—H⋯Cl ammonium-to-chloride hydrogen bonds and O—H⋯Cl hydrogen bonds combine to generate rings with graph-set notation 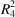 (20). The two rings and chains combine to give ladder chains along [010], Fig. 2 ▸.

The most closely related structure to the title compound is the two-to-one 4-HO-DPT-to-fumarate salt, which crystallizes as a tetra­hydrate (WUCGAF; Chadeayne *et al.*, 2019*b* ▸). There are six other structures of 4-hy­droxy-substituted tryptamines that have been reported. These are the active metabolite of psilocybin - psilocin, or 4-hy­droxy-*N*,*N*-di­methyl­tryptamine (PSILIN; Petcher & Weber, 1974 ▸), the active metabolite of baeocystin-norpsilocin, or 4-hy­droxy-*N*-methyl­tryptamine, which has been reported as its freebase (CCDC 1992279; Chadeayne *et al.*, 2020*b* ▸) and its fumarate salt (CCDC 1992278; Chadeayne *et al.*, 2020*b* ▸), and the active metabolite of aeruginascin − 4-hy­droxy-*N*,*N*,*N*-tri­methyl­tryptamine (XUXFAA; Chadeayne, Pham, Reid *et al.*, 2020 ▸) which is reported as its iodide salt. The structure of the synthetic psychedelic 4-HO-MiPT has also been reported as its one-to-one hydro­fumarate salt (RONSUL; Chadeayne *et al.*, 2019*a* ▸) and as its two-to-one fumarate salt (CCDC 1987588; Chadeayne *et al.*, 2 2020*a* ▸).

## Synthesis and crystallization

Freebase 4-hy­droxy-*N*,*N*-di-*n*-propyl­tryptamine (50 mg, 0.19 mmol) was dissolved in di­chloro­methane, and 160 µ*L* of hydorochloric acid (1.25 *M* in ethanol, 0.20 mmol) were added with stirring at room temperature. The mixture was stirred for 30 minutes, resulting in a white precipitate which was isolated *via* vacuum filtration and washed with diethyl ether to yield 28 mg of the salt. A second crop was collected by concentrating and cooling the filtrate to give another 13 mg of the salt (73% yield). Crystals suitable for X-ray diffraction studies were grown by the slow evaporation of a methyl­ene chloride/methanol mixture. The sample was analyzed by nuclear magnetic resonance. ^1^H NMR (400 MHz, D_2_O): *δ* 7.07 (*s*, 1 H, Ar*H*), 7.04–6.96 (*m*, 2 H, Ar*H*), 6.47 (*dd*, *J* = 6.1, 2.4 Hz, 1 H, Ar*H*), 3.42–3.30 (*m*, 2 H, C*H*_2_), 3.20–3.07 (*m*, 2 H, C*H*_2_), 3.00 (*dd*, *J* = 10.1, 6.4 Hz, 4 H, C*H*_2_), 1.60 (*h*, *J* = 6.8 Hz, 4 H, C*H*_2_), 0.84 (*t*, *J* = 7.4 Hz, 6 H, C*H*_3_); ^13^C NMR (100 MHz, D_2_O): *δ* 149.9 (Ar*C*), 138.8 (Ar*C*), 123.3 (Ar*C*), 123.2 (Ar*C*), 115.9 (Ar*C*), 108.5 (Ar*C*), 104.5 (Ar*C*), 103.7 (Ar*C*), 54.7 (Ak*C*), 53.7 (Ak*C*), 21.2 (Ak*C*), 16.8 (Ak*C*), 10.0 (Ak*C*).

## Refinement

Crystal data, data collection and structure refinement details are summarized in Table 2 ▸.

## Supplementary Material

Crystal structure: contains datablock(s) I. DOI: 10.1107/S2414314620015461/sj4218sup1.cif

Structure factors: contains datablock(s) I. DOI: 10.1107/S2414314620015461/sj4218Isup2.hkl

Supporting information file. DOI: 10.1107/S2414314620015461/sj4218Isup3.cml

CCDC reference: 2045782

Additional supporting information:  crystallographic information; 3D view; checkCIF report

## Figures and Tables

**Table 1:** Hydrogen-bond geometry (Å, °)

*D*—H⋯*A*	*D*—H	H⋯*A*	*D*⋯*A*	*D*—H⋯*A*
N1—H1⋯Cl1^i^	0.88 (1)	2.40 (1)	3.2766 (18)	176 (2)
N2—H2⋯Cl1^ii^	0.98 (1)	2.15 (1)	3.1126 (17)	169 (2)
O1—H1*A*⋯Cl1	0.86 (1)	2.26 (1)	3.1068 (15)	170 (2)

Symmetry codes: (i) 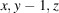 ; (ii) 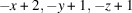 .

**Table 2:** Experimental details

Crystal data	
Chemical formula	C_16_H_25_N_2_O^+^·Cl^−^
*M*_r_	296.83
Crystal system, space group	Triclinic, *P* 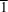
Temperature (K)	273
*a*, *b*, *c* (Å)	7.860 (3), 10.439 (4), 11.713 (5)
α, β, γ (°)	76.236 (14), 73.653 (13), 68.852 (12)
*V* (Å^3^)	850.0 (6)
*Z*	2
Radiation type	Mo *K*α
μ (mm^−1^)	0.22
Crystal size (mm)	0.15 × 0.10 × 0.10

Data collection	
Diffractometer	Bruker D8 Venture CMOS
Absorption correction	Multi-scan (*SADABS*; Bruker, 2018 ▸)
*T*_min_, *T*_max_	0.720, 0.745
No. of measured, independent and observed [*I* > 2σ(*I*)] reflections	27008, 3243, 2756
*R*_int_	0.066
(sin θ/λ)_max_ (Å^−1^)	0.616

Refinement	
*R*[*F* ^2^ > 2σ(*F* ^2^)], *wR*(*F* ^2^), *S*	0.036, 0.103, 1.03
No. of reflections	3243
No. of parameters	192
No. of restraints	3
H-atom treatment	H atoms treated by a mixture of independent and constrained refinement
Δρ_max_, Δρ_min_ (e Å^−3^)	0.43, −0.40

Computer programs: *APEX3* and *SAINT* (Bruker, 2018 ▸), *SHELXT2014* (Sheldrick, 2015*a* ▸), *SHELXL2018* (Sheldrick, 2015*b* ▸), *OLEX2* (Dolomanov *et al.*, 2009 ▸) and *publCIF* (Westrip, 2010 ▸).

## full crystallographic data

### Crystal data

**Table:**

C_16_H_25_N_2_O^+^·Cl^−^	*Z* = 2
*M**_r_* = 296.83	*F*(000) = 320
Triclinic, *P*1	*D*_x_ = 1.160 Mg m^−^^3^
*a* = 7.860 (3) Å	Mo *K*α radiation, λ = 0.71073 Å
*b* = 10.439 (4) Å	Cell parameters from 9206 reflections
*c* = 11.713 (5) Å	θ = 2.6–25.8°
α = 76.236 (14)°	µ = 0.22 mm^−^^1^
β = 73.653 (13)°	*T* = 273 K
γ = 68.852 (12)°	Block, colourless
*V* = 850.0 (6) Å^3^	0.15 × 0.10 × 0.10 mm

### Data collection

**Table:**

Bruker D8 Venture CMOS diffractometer	2756 reflections with *I* > 2σ(*I*)
φ and ω scans	*R*_int_ = 0.066
Absorption correction: multi-scan (SADABS; Bruker, 2018)	θ_max_ = 26.0°, θ_min_ = 2.6°
*T*_min_ = 0.720, *T*_max_ = 0.745	*h* = −9→9
27008 measured reflections	*k* = −12→12
3243 independent reflections	*l* = −14→14

### Refinement

**Table:**

Refinement on *F*^2^	3 restraints
Least-squares matrix: full	Hydrogen site location: mixed
*R*[*F*^2^ > 2σ(*F*^2^)] = 0.036	H atoms treated by a mixture of independent and constrained refinement
*wR*(*F*^2^) = 0.103	*w* = 1/[σ^2^(*F*_o_^2^) + (0.0541*P*)^2^ + 0.1743*P*] where *P* = (*F*_o_^2^ + 2*F*_c_^2^)/3
*S* = 1.03	(Δ/σ)_max_ = 0.001
3243 reflections	Δρ_max_ = 0.43 e Å^−^^3^
192 parameters	Δρ_min_ = −0.40 e Å^−^^3^

### Special details

**Table:**

Geometry. All esds (except the esd in the dihedral angle between two l.s. planes) are estimated using the full covariance matrix. The cell esds are taken into account individually in the estimation of esds in distances, angles and torsion angles; correlations between esds in cell parameters are only used when they are defined by crystal symmetry. An approximate (isotropic) treatment of cell esds is used for estimating esds involving l.s. planes.

### Fractional atomic coordinates and isotropic or equivalent isotropic displacement parameters (Å^2^)

**Table:**

	*x*	*y*	*z*	*U*_iso_*/*U*_eq_	
Cl1	0.84351 (5)	0.74832 (4)	0.23642 (4)	0.04716 (14)	
N1	0.7748 (2)	0.03222 (14)	0.34007 (16)	0.0578 (4)	
H1	0.790 (3)	−0.0454 (14)	0.3157 (19)	0.069*	
N2	0.75749 (17)	0.34070 (13)	0.71443 (11)	0.0422 (3)	
H2	0.8748 (18)	0.314 (2)	0.7409 (18)	0.063*	
O1	0.77173 (17)	0.46842 (11)	0.35885 (10)	0.0506 (3)	
H1A	0.797 (3)	0.5412 (16)	0.3168 (19)	0.076*	
C1	0.7077 (2)	0.05956 (16)	0.45593 (18)	0.0531 (4)	
H1B	0.668481	−0.001309	0.521524	0.064*	
C2	0.7068 (2)	0.18915 (15)	0.46130 (15)	0.0416 (3)	
C3	0.77946 (19)	0.24542 (15)	0.34010 (14)	0.0385 (3)	
C4	0.8114 (2)	0.37253 (16)	0.28523 (14)	0.0413 (3)	
C5	0.8792 (2)	0.3950 (2)	0.16234 (16)	0.0547 (4)	
H5	0.898048	0.479641	0.125550	0.066*	
C6	0.9198 (3)	0.2913 (2)	0.09275 (18)	0.0657 (5)	
H6	0.966338	0.308406	0.010282	0.079*	
C7	0.8929 (3)	0.1653 (2)	0.14258 (18)	0.0636 (5)	
H7	0.921712	0.096793	0.095694	0.076*	
C8	0.8204 (2)	0.14383 (16)	0.26682 (17)	0.0480 (4)	
C9	0.6397 (2)	0.25770 (17)	0.57189 (14)	0.0437 (4)	
H9A	0.559043	0.212172	0.633105	0.052*	
H9B	0.567594	0.354321	0.552523	0.052*	
C10	0.8050 (2)	0.24920 (16)	0.62024 (14)	0.0437 (4)	
H10A	0.860211	0.153568	0.654519	0.052*	
H10B	0.898501	0.274491	0.553374	0.052*	
C11	0.7172 (3)	0.49232 (17)	0.65963 (16)	0.0518 (4)	
H11A	0.814555	0.501143	0.588662	0.062*	
H11B	0.599814	0.523916	0.633915	0.062*	
C12	0.7061 (3)	0.58502 (19)	0.74365 (18)	0.0605 (5)	
H12A	0.808731	0.542065	0.784928	0.073*	
H12B	0.590170	0.596278	0.803782	0.073*	
C13	0.7149 (3)	0.72656 (19)	0.6756 (2)	0.0640 (5)	
H13A	0.829874	0.715522	0.616365	0.096*	
H13B	0.708833	0.783488	0.730965	0.096*	
H13C	0.611458	0.770155	0.636457	0.096*	
C14	0.6079 (2)	0.31594 (18)	0.82147 (15)	0.0520 (4)	
H14A	0.494348	0.334255	0.794356	0.062*	
H14B	0.581895	0.381292	0.875563	0.062*	
C15	0.6587 (3)	0.1708 (2)	0.88982 (18)	0.0638 (5)	
H15A	0.783677	0.144610	0.903678	0.077*	
H15B	0.657852	0.106282	0.842690	0.077*	
C16	0.5201 (4)	0.1627 (3)	1.0100 (2)	0.0906 (8)	
H16A	0.521750	0.226249	1.056699	0.136*	
H16B	0.554431	0.069863	1.053005	0.136*	
H16C	0.396891	0.186970	0.996033	0.136*	

### Atomic displacement parameters (Å^2^)

**Table:**

	*U*^11^	*U*^22^	*U*^33^	*U*^12^	*U*^13^	*U*^23^
Cl1	0.0460 (2)	0.0406 (2)	0.0602 (3)	−0.01650 (16)	−0.00926 (17)	−0.01564 (16)
N1	0.0652 (9)	0.0341 (7)	0.0837 (11)	−0.0083 (6)	−0.0303 (8)	−0.0213 (7)
N2	0.0399 (7)	0.0438 (7)	0.0419 (7)	−0.0115 (5)	−0.0077 (5)	−0.0087 (5)
O1	0.0656 (7)	0.0389 (6)	0.0501 (7)	−0.0234 (5)	−0.0027 (5)	−0.0128 (5)
C1	0.0553 (9)	0.0371 (8)	0.0727 (12)	−0.0163 (7)	−0.0253 (9)	−0.0027 (8)
C2	0.0378 (7)	0.0364 (7)	0.0535 (9)	−0.0114 (6)	−0.0144 (6)	−0.0070 (6)
C3	0.0332 (7)	0.0372 (7)	0.0477 (8)	−0.0076 (6)	−0.0108 (6)	−0.0145 (6)
C4	0.0375 (7)	0.0405 (8)	0.0473 (8)	−0.0106 (6)	−0.0076 (6)	−0.0136 (6)
C5	0.0570 (10)	0.0615 (10)	0.0472 (9)	−0.0235 (8)	−0.0070 (8)	−0.0091 (8)
C6	0.0657 (11)	0.0873 (14)	0.0450 (10)	−0.0218 (10)	−0.0056 (8)	−0.0230 (10)
C7	0.0618 (11)	0.0694 (12)	0.0637 (12)	−0.0038 (9)	−0.0173 (9)	−0.0399 (10)
C8	0.0431 (8)	0.0411 (8)	0.0640 (10)	−0.0028 (7)	−0.0197 (7)	−0.0223 (7)
C9	0.0377 (8)	0.0469 (8)	0.0467 (9)	−0.0154 (6)	−0.0054 (6)	−0.0083 (7)
C10	0.0375 (7)	0.0460 (8)	0.0458 (8)	−0.0088 (6)	−0.0064 (6)	−0.0135 (7)
C11	0.0578 (10)	0.0456 (9)	0.0511 (10)	−0.0148 (8)	−0.0137 (8)	−0.0056 (7)
C12	0.0719 (12)	0.0498 (10)	0.0640 (11)	−0.0216 (9)	−0.0160 (9)	−0.0110 (8)
C13	0.0618 (11)	0.0491 (10)	0.0826 (14)	−0.0203 (9)	−0.0180 (10)	−0.0054 (9)
C14	0.0510 (9)	0.0567 (10)	0.0459 (9)	−0.0181 (8)	−0.0019 (7)	−0.0120 (7)
C15	0.0807 (13)	0.0597 (11)	0.0542 (11)	−0.0317 (10)	−0.0108 (9)	−0.0038 (8)
C16	0.126 (2)	0.0990 (17)	0.0581 (13)	−0.0693 (17)	0.0035 (13)	−0.0071 (12)

### Geometric parameters (Å, º)

**Table:**

N1—H1	0.880 (9)	C9—H9B	0.9700
N1—C1	1.369 (3)	C9—C10	1.524 (2)
N1—C8	1.369 (3)	C10—H10A	0.9700
N2—H2	0.980 (9)	C10—H10B	0.9700
N2—C10	1.510 (2)	C11—H11A	0.9700
N2—C11	1.512 (2)	C11—H11B	0.9700
N2—C14	1.502 (2)	C11—C12	1.502 (3)
O1—H1A	0.856 (10)	C12—H12A	0.9700
O1—C4	1.3700 (19)	C12—H12B	0.9700
C1—H1B	0.9300	C12—C13	1.518 (3)
C1—C2	1.366 (2)	C13—H13A	0.9600
C2—C3	1.441 (2)	C13—H13B	0.9600
C2—C9	1.501 (2)	C13—H13C	0.9600
C3—C4	1.407 (2)	C14—H14A	0.9700
C3—C8	1.413 (2)	C14—H14B	0.9700
C4—C5	1.382 (2)	C14—C15	1.504 (3)
C5—H5	0.9300	C15—H15A	0.9700
C5—C6	1.401 (3)	C15—H15B	0.9700
C6—H6	0.9300	C15—C16	1.524 (3)
C6—C7	1.371 (3)	C16—H16A	0.9600
C7—H7	0.9300	C16—H16B	0.9600
C7—C8	1.401 (3)	C16—H16C	0.9600
C9—H9A	0.9700		
			
C1—N1—H1	126.0 (15)	N2—C10—H10A	108.6
C1—N1—C8	109.42 (14)	N2—C10—H10B	108.6
C8—N1—H1	124.6 (15)	C9—C10—H10A	108.6
C10—N2—H2	103.4 (12)	C9—C10—H10B	108.6
C10—N2—C11	111.53 (13)	H10A—C10—H10B	107.6
C11—N2—H2	105.8 (12)	N2—C11—H11A	108.8
C14—N2—H2	108.7 (12)	N2—C11—H11B	108.8
C14—N2—C10	114.06 (13)	H11A—C11—H11B	107.7
C14—N2—C11	112.56 (13)	C12—C11—N2	113.84 (14)
C4—O1—H1A	109.4 (16)	C12—C11—H11A	108.8
N1—C1—H1B	124.9	C12—C11—H11B	108.8
C2—C1—N1	110.22 (16)	C11—C12—H12A	109.4
C2—C1—H1B	124.9	C11—C12—H12B	109.4
C1—C2—C3	106.02 (15)	C11—C12—C13	111.01 (17)
C1—C2—C9	126.16 (16)	H12A—C12—H12B	108.0
C3—C2—C9	127.82 (13)	C13—C12—H12A	109.4
C4—C3—C2	134.47 (14)	C13—C12—H12B	109.4
C4—C3—C8	118.35 (15)	C12—C13—H13A	109.5
C8—C3—C2	107.17 (14)	C12—C13—H13B	109.5
O1—C4—C3	116.94 (14)	C12—C13—H13C	109.5
O1—C4—C5	123.52 (15)	H13A—C13—H13B	109.5
C5—C4—C3	119.54 (15)	H13A—C13—H13C	109.5
C4—C5—H5	119.8	H13B—C13—H13C	109.5
C4—C5—C6	120.47 (17)	N2—C14—H14A	108.8
C6—C5—H5	119.8	N2—C14—H14B	108.8
C5—C6—H6	119.0	N2—C14—C15	113.64 (15)
C7—C6—C5	121.98 (18)	H14A—C14—H14B	107.7
C7—C6—H6	119.0	C15—C14—H14A	108.8
C6—C7—H7	121.3	C15—C14—H14B	108.8
C6—C7—C8	117.46 (16)	C14—C15—H15A	109.6
C8—C7—H7	121.3	C14—C15—H15B	109.6
N1—C8—C3	107.18 (16)	C14—C15—C16	110.23 (18)
N1—C8—C7	130.64 (16)	H15A—C15—H15B	108.1
C7—C8—C3	122.18 (17)	C16—C15—H15A	109.6
C2—C9—H9A	109.6	C16—C15—H15B	109.6
C2—C9—H9B	109.6	C15—C16—H16A	109.5
C2—C9—C10	110.36 (12)	C15—C16—H16B	109.5
H9A—C9—H9B	108.1	C15—C16—H16C	109.5
C10—C9—H9A	109.6	H16A—C16—H16B	109.5
C10—C9—H9B	109.6	H16A—C16—H16C	109.5
N2—C10—C9	114.75 (12)	H16B—C16—H16C	109.5
			
N1—C1—C2—C3	−0.34 (18)	C4—C3—C8—N1	−178.94 (13)
N1—C1—C2—C9	178.71 (14)	C4—C3—C8—C7	0.6 (2)
N2—C11—C12—C13	165.62 (15)	C4—C5—C6—C7	0.6 (3)
N2—C14—C15—C16	168.13 (17)	C5—C6—C7—C8	0.8 (3)
O1—C4—C5—C6	178.91 (16)	C6—C7—C8—N1	178.09 (18)
C1—N1—C8—C3	−0.20 (18)	C6—C7—C8—C3	−1.4 (3)
C1—N1—C8—C7	−179.73 (17)	C8—N1—C1—C2	0.34 (19)
C1—C2—C3—C4	178.89 (16)	C8—C3—C4—O1	−179.49 (13)
C1—C2—C3—C8	0.21 (16)	C8—C3—C4—C5	0.7 (2)
C1—C2—C9—C10	101.54 (18)	C9—C2—C3—C4	−0.1 (3)
C2—C3—C4—O1	1.9 (2)	C9—C2—C3—C8	−178.82 (14)
C2—C3—C4—C5	−177.84 (15)	C10—N2—C11—C12	−168.69 (14)
C2—C3—C8—N1	−0.01 (17)	C10—N2—C14—C15	61.10 (19)
C2—C3—C8—C7	179.57 (15)	C11—N2—C10—C9	−72.81 (17)
C2—C9—C10—N2	167.42 (13)	C11—N2—C14—C15	−170.53 (15)
C3—C2—C9—C10	−79.62 (19)	C14—N2—C10—C9	56.08 (18)
C3—C4—C5—C6	−1.3 (3)	C14—N2—C11—C12	61.62 (19)

### Hydrogen-bond geometry (Å, º)

**Table:**

*D*—H···*A*	*D*—H	H···*A*	*D*···*A*	*D*—H···*A*
N1—H1···Cl1^i^	0.88 (1)	2.40 (1)	3.2766 (18)	176 (2)
N2—H2···Cl1^ii^	0.98 (1)	2.15 (1)	3.1126 (17)	169 (2)
O1—H1*A*···Cl1	0.86 (1)	2.26 (1)	3.1068 (15)	170 (2)

Symmetry codes: (i) *x*, *y*−1, *z*; (ii) −*x*+2, −*y*+1, −*z*+1.
